# The Effect of Age, Hypertension, and Overweight on Arterial Stiffness Assessed Using Carotid Wall Echo-Tracking in Childhood and Adolescence

**DOI:** 10.3390/life14030300

**Published:** 2024-02-23

**Authors:** Tomas Jurko, Michal Mestanik, Eva Jurkova, Kamil Zelenak, Eva Klaskova, Alexander Jurko

**Affiliations:** 1 Clinic of Neonatology, Jessenius Faculty of Medicine in Martin (JFM CU), Comenius University in Bratislava, University Hospital Martin, Kollarova 2, 03659 Martin, Slovakia; jurko3@uniba.sk; 2 Department of Radiodiagnostics, DONsP L. N. Jege Hospital Dolny Kubin, Nemocnicna 1944/10, 02614 Dolny Kubin, Slovakia; mestanik@gmail.com; 3 Pediatric Cardiology Clinic, Kollarova 13, 03601 Martin, Slovakia; ltvsro@gmail.com; 4 Clinic of Children and Adolescents, Jessenius Faculty of Medicine in Martin (JFM CU), Comenius University in Bratislava, University Hospital Martin, Kollarova 2, 03659 Martin, Slovakia; malicherova3@uniba.sk; 5 Clinic of Radiology, Jessenius Faculty of Medicine in Martin (JFM CU), Comenius University in Bratislava, University Hospital Martin, Kollarova 2, 03659 Martin, Slovakia; kamil.zelenak@uniba.sk; 6 Department of Pediatrics, Faculty of Medicine and Dentistry, Palacky University, University Hospital Olomouc, Zdravotniku 248/7, 77900 Olomouc, Czech Republic

**Keywords:** arterial stiffness, carotid wall echo-tracking, blood pressure, essential hypertension, white coat hypertension, childhood, adolescence, overweight, obesity, reference values

## Abstract

Arterial stiffness represents an independent predictor of the risk of subsequent cardiovascular events. Early identification of high-risk individuals is necessary for effective prevention and targeted interventions. Carotid wall echo-tracking is a modern method for an accurate evaluation of the structural and functional properties of carotid arteries. This study aimed to assess age and sex-specific reference values of the echo-tracking parameters of carotid stiffness in 400 healthy children and adolescents and to evaluate the potential early effect of elevated blood pressure and overweight in 69 overweight normotensives, 45 white coat hypertensives, and 44 essential hypertensives. Stiffness index β, pressure–strain elastic modulus (Ep), arterial compliance (AC), and pulse wave velocity β (PWV β) were evaluated using Aloka ProSound F75. Both white coat and essential hypertension were associated with impaired carotid wall properties with the greatest effect on Ep, followed by PWV β, index β, and AC. The excess weight showed a weaker effect on Ep and PWV β. This is the first study to compare the effects of white coat and essential hypertension on carotid arterial stiffness assessed using the echo-tracking technique in childhood and adolescence with direct application of pediatric reference values specific to age and sex.

## 1. Introduction

Cardiovascular diseases (CVD) are the leading cause of morbidity, mortality, and health costs in developed and developing countries. Strategies to reduce the CVD burden are often focused on the adult population; however, initial pathophysiological alterations are already present in childhood and depend on exposure to several risk factors [1]. Among these, children with overweight and prehypertensive or hypertensive blood pressure levels have a markedly increased risk of developing cardiovascular events early at 40 years of age [2,3]. Therefore, the selection of appropriate diagnostic approaches for the detection of subclinical cardiovascular damage in the pediatric population is a key step toward the early identification of high-risk individuals and the reduction in morbidity in later life. 

Arterial stiffening is one of the earliest manifestations of the adverse effects of cardiovascular risk factors on arterial structure and function, leading to reduced arterial expansion and recoil capacity in response to pressure changes [4]. As a result, elevated pulse pressure leads to left ventricular overload, reduced coronary perfusion, and damage to the microcirculation of various organs [5]. Stiffening of the arterial wall progresses non-uniformly and preferentially affects the proximal segments, which contain more elastic elements [4,6]. Evaluation of local arterial stiffness at the carotid artery level offers a unique possibility of detecting initial arteriosclerotic alterations in a well-accessible location. Carotid stiffness, particularly load-dependent stiffening due to elevated blood pressure (BP), is considered an independent predictor of incident CVD [7]. Moreover, increased arterial stiffness in adolescence is nowadays discussed as a potential novel risk factor for a broader spectrum of hypertensive and metabolic diseases in young adulthood [8].

Modern methods of echo-tracking of the carotid wall use high-resolution radiofrequency signal analysis to evaluate the pressure–diameter relationships of the rapid movements of the arterial wall during the cardiac cycle [9]. This results in a more precise calculation of the local carotid stiffness—β-stiffness index, pressure–strain elastic modulus (Peterson’s) (Ep), arterial compliance (AC), and pulse wave velocity β (PWV β). Regarding the effect of hypertension and overweight on carotid stiffness in children and adolescents, previous studies revealed contradictory findings. Although Ep was evaluated using traditional B and M mode ultrasound and was found to increase in adolescents and young adults with prehypertension and essential hypertension [10], no significant differences in parameters β, Ep, and PWV β were found between hypertensive and normotensive adolescents when evaluated using the echo-tracking method [11]. Studies on the effect of body composition appear to be more consistent, revealing increased β, Ep, and PWV β in obese children compared to their non-obese counterparts [12,13]. However, in non-obese children, carotid distensibility, incremental modulus, and β were affected by BP, but not body mass index [14]. Furthermore, there is a lack of data on the effect of white coat hypertension, which is a common diagnosis in pediatric patients referred for the evaluation of elevated office BP [15]. 

Therefore, our objective was to (1) collect reference values of the carotid wall stiffness indices β, Ep, AC, and PWV β evaluated using the echo-tracking method in 400 healthy children and adolescents aged 7–18 years, and (2) study the potential effect of pediatric white coat hypertension, essential hypertension, and overweight on these parameters.

## 2. Materials and Methods

### 2.1. Subjects

The participants in this cross-sectional study were prospectively recruited from local elementary schools, high schools, and clinical offices on a voluntary basis. They were invited to participate in the study and received information about the aim of the study, description of the examination procedure, and information about the approval by the Ethics Committee. 

The study was conducted in accordance with the Declaration of Helsinki and was approved by the Ethics Committee of Jessenius Faculty of Medicine in Martin, Comenius University in Bratislava, approval code EK 1571/2014. All children and their parents/legal representatives signed informed consent prior to participation in the study.

**Figure 1:**
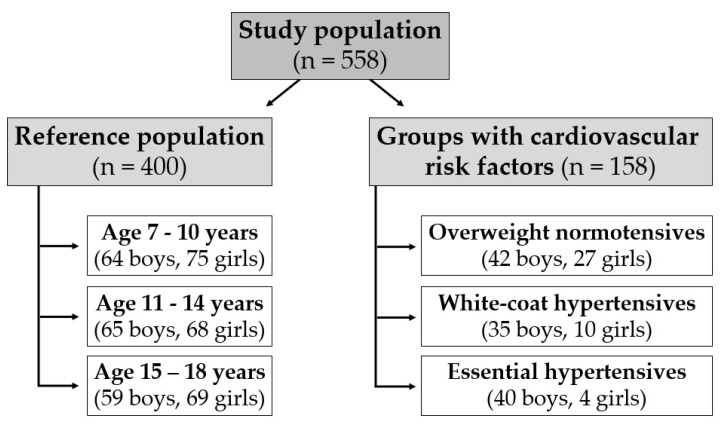
Flow chart of study population—reference population of healthy non-overweight normotensive participants and the groups of participants with cardiovascular risk factors—overweight, white coat hypertension, and essential hypertension.

A total of 558 participants aged 7–18 years were divided into four groups: reference population of 400 healthy non-overweight normotensive participants, 69 otherwise healthy overweight normotensives, 45 white coat hypertensives, and 44 essential hypertensives (Figure 1). 

The inclusion criteria were age between 7 and 18 years, no known diseases and normal weight in reference population, confirmed diagnosis of overweight, white coat hypertension, and essential hypertension in the respective groups (specified in Section 2.1.1 and Section 2.1.2). The following exclusion criteria were strictly applied prior to enrolling children in this study: smoking, acute illness in the past two weeks, chronic cardiovascular, respiratory, endocrine, neurological, metabolic, or infectious diseases or mental disorders, and use of medicaments or supplements with the potential effect on the cardiovascular or autonomic nervous systems.

#### 2.1.1. Anthropometric Measures and the Diagnosis of Overweight

Weight (in kilograms) and height (in meters) were measured, and body mass index (BMI) was calculated as body weight divided by height squared. Normal weight and overweight were defined according to the Extended International Obesity Task Force (IOTF) body mass index cut-off points for thinness, overweight, and obesity using age and sex-specific body mass index (BMI) cut-off points, which correspond to the adult BMI range between 18.5 and 25 kg m^−2^ for normal weight, and the threshold of 25 kg m^−2^ for overweight [16]. 

#### 2.1.2. Diagnosis of Hypertension

In participants with elevated BP, the diagnosis of essential (primary) hypertension and white coat hypertension was confirmed by a specialist in pediatric cardiology following the recommendations for the management of high BP in children and adolescents [17]. The diagnosis of hypertension was determined as systolic BP and/or diastolic BP ≥ 95th percentile of the age- and sex-specific reference values corrected for height [18]. The auscultatory method was used for BP measurements at three consecutive visits to the clinical office. Afterwards, 24 h ambulatory BP monitoring was used to differentiate between the diagnoses of essential and white coat hypertension, which was characterized by BP ≥ 95th percentile of the reference values in the clinical conditions but <90th percentile in the home environment [18]. Secondary cause of elevated BP was ruled out for all participants.

### 2.2. Protocol

The examinations were carried out between 8.00 and 11.00 a.m. under standard conditions (temperature of the room 22 °C, minimalization of the stimuli). Before examination, BP was measured and participants were rested in the supine position for 15 min. Vascular ultrasound examinations were performed using the Aloka ProSound F75 ultrasound system (Aloka Co., Ltd., Tokyo, Japan) with integrated echo-tracking technology and a spatial resolution of 5–16 MHz in the common carotid artery (CCA) on both sides. The multifrequency linear array probe was placed longitudinally over the distal CCA and the site of the measurement was located about a centimeter proximal to the carotid sinus. Independently steered ultrasound beams were used for evaluation of diameter change and blood flow velocity. The changes in diameter were measured as the differences between the displacement waveforms of the anterior and posterior walls, with the cursors manually set at the intima–media boundaries. Blood flow velocity was measured at the center of the CCA diameter using automatically positioned echo-tracking gates. A minimum of five consecutive cardiac cycles was used for the analysis. Simultaneously, brachial BP values were acquired for the calibration of the diameter waveforms. 

### 2.3. Evaluated Parameters of Carotid Wall Echo-Tracking

In this study, four physiological characteristics of the CCA wall were analyzed—β, Ep, AC, and PWV β. 

The stiffness index β was calculated using the following equation:

stiffness indexβ=In(Ps/Pd)/[(Ds−Dd)/Dd],

where Ps = systolic BP, Pd = diastolic BP, Ds = arterial systolic diameter, Dd = arterial diastolic diameter.

The pressure–strain elastic modulus (Peterson’s) was calculated from the changes in CCA diameter and BP.

Ep=(Ps−Pd)/[(Ds−Dd)/Dd].

Arterial compliance (AC) was calculated from the arterial cross area and BP:

$$\mathrm{AC}=\pi \left(Ds^{2}-Dd^{2}\right)/4\left(\mathrm{Ps}-\mathrm{Pd}\right)\;$$

Pulse wave velocity β (PWV β) was calculated from the time delay between the two adjacent distension waveforms of the water hammer equation for forward traveling waves using the β-stiffness parameter.

The increase in the indices β, EP, and PWV β and the decrease in the parameter AC reflects the stiffening of the vessel wall. The assessment of these parameters using Aloka ProSound echo-tracking method was found to be characterized by good intraobserver variability (3.8%, 4.9%, 6.2%, and 5.5% for the parameters β, Ep, AC, and PWV β, respectively), good interobserver variability (7.2%, 7.0%, 6.6%, and 6.3% for β, Ep, AC, and PWV β, respectively), and good test–retest variability (5.2%, 10.1%, 5.9%, and 4.3% for β, Ep, AC, and PWV β, respectively) [19]. For β, EP, and PWV β, the higher values from the left and right sides were included in the statistical analyses. For AC, the lower values of the left and right sides were used.

### 2.4. Statistical Analysis

The statistical program SYSTAT 10 (Cranes Software International Ltd., Troy, MI, USA) was used for the data analysis. The normality of the distribution was evaluated using the Shapiro–Wilk test. 

Comparison of differences in clinical data and parameters β, Ep, AC, and PWV β between age-specific groups (7–10, 11–14, and 15–18 years) of the reference population of 400 non-overweight normotensive participants was performed using the analysis of variance (ANOVA) and post hoc Holm–Sidak test for data with normal distribution and equal variance or using Kruskal–Wallis test with post hoc Dunn’s multiple pairwise comparison for parameters with non-normal distribution or unequal variance. Sex differences were analyzed using two-sample Student’s *t* test for data with normal distribution and using the Mann–Whitney U test for variables with nonnormal distribution.

The reference values of the parameters β, Ep, AC, and PWV β were assessed as percentile values in the sex- and age-specific groups of healthy non-overweight normotensive participants. The carotid wall stiffness parameters were compared in overweight and hypertensive subjects with the corresponding reference ranges. The values of β, Ep, and PWV β higher than the 95th percentile and AC lower than the 5th percentile were considered abnormal. 

The potential effects of age, sex, overweight, and hypertension on the carotid wall stiffness indices were analyzed in the entire studied population, including 400 non-overweight normotensives, 69 overweight normotensives, 45 white coat hypertensives, and 44 essential hypertensives. Linear regression modeling was used with the parameters β, Ep, AC, and PWV β as dependent variables and age, male sex, overweight, white coat hypertension, and essential hypertension as candidate predictors. The parameters male sex, overweight, white coat hypertension, and essential hypertension were included as dummy-coded categorical variables. The presence of multicollinearity was checked using the variance inflation factor (VIF) assessment.

The probabilities of *p* < 0.05 were considered significant.

## 3. Results

### 3.1. Characteristics of the Evaluated Groups

The clinical data of the age- and sex-specific groups of the reference population of 400 healthy non-overweight normotensive subjects are summarized in Table 1. The age group (7–10, 11–14, and 15–18 years) showed significant effect on BMI, systolic BP, and heart rate for both males (*F*[2] = 104.59, *p* < 0.001; *χ*^2^[2] = 79.84, *p* < 0.001; *χ*^2^[2] = 30.20, *p* < 0.001; respectively), and females (*χ*^2^[2] = 127.51, *p* < 0.001; *F*[2] = 26.30, *p* < 0.001; *χ*^2^[2] = 15.90, *p* < 0.001; respectively). The effect on diastolic BP was significant only for females (*F*[2] = 5.62, *p* = 0.004).

Post hoc analysis revealed a progressive increase in BMI and systolic BP and a progressive decrease in heart rate from the age of 7 to 10 to 15 to 18 years. In males, BMI and systolic BP were significantly higher in the group aged 11–14 years compared to subjects aged 7–10 years and in the group aged 15–18 years compared to subjects aged 7–10 and 11–14 years (*p* < 0.001 for all comparisons). Heart rate was significantly lower in the group aged 11–14 years compared to subjects aged 7–10 years (*p* = 0.012) and in the group aged 15–18 compared to subjects aged 7–10 and 11–14 years (*p* < 0.001, *p* = 0.002; respectively).

In females, BMI and systolic BP were significantly higher in the group aged 11–14 years compared to subjects aged 7–10 years (*p* < 0.001, *p* = 0.021; respectively), and in the group aged 15–18 years compared to subjects aged 7–10 and 11–14 years (*p* < 0.001 for all comparisons). Heart rate was significantly lower in the group aged 11–14 years compared to subjects aged 7–10 years (*p* = 0.015) and in the group aged 15–18 compared to subjects aged 7–10 and 11–14 years (*p* < 0.001 for both). Diastolic BP was significantly higher in subjects aged 15–18 years compared to those aged 11–14 years (*p* < 0.001). The effect of sex was significant for systolic BP in the age group 15–18 years (*p* < 0.001) and for heart rate in the groups aged 7–10 to 15–18 years (*p* = 0.015, *p* = 0.020, *p* < 0.001; respectively).

The clinical data of the overweight normotensive subjects, subjects with white coat hypertension, and subjects with essential hypertension are summarized in Table 2.

### 3.2. Reference Values of the Stiffness Parameters of the Carotid Wall in the Sex- and Age-Specific Groups

The age group (7–10, 11–14, and 15–18 years) showed significant effect on the carotid stiffness indices β, Ep, AC and PWV β for both males (χ^2^[2] = 89.16, *p* < 0.001; χ^2^[2] = 116.96, *p* < 0.001; χ^2^[2] = 59.30, *p* < 0.001; χ^2^[2] = 102.85, *p* < 0.001; respectively), and females (χ^2^[2] = 91.08, *p* < 0.001; F[2] = 99.79, *p* < 0.001; χ^2^[2] = 31.54, *p* < 0.001; F[2] = 91.37, *p* < 0.001; respectively). In males, post hoc analysis revealed a progressive increase in the parameters β, Ep, and PWV β from the age of 7 to 10 to 15 to 18 years. The group aged 11–14 years showed significantly higher values compared to subjects aged 7–10 years (*p* < 0.001 for all comparisons). The subjects aged 15–18 showed significantly higher values compared to those aged 7–10 and 11–14 years (*p* < 0.001 for all comparisons). Similarly, a progressive decrease in AC was found in males at the age of 11–14 years compared to those aged 7–10 years and in the age group 15–18 years compared to 7–10 and 11–14 years (*p* < 0.001 for all comparisons). Similar results were found in females with a progressive increase in β, Ep, and PWV β from 7 to 10 to 15 to 18 years of age. All three indices showed significantly higher values in the age group 11–14 years compared to 7–10 years (*p* < 0.001 for all comparisons) and in the age group 15–18 years compared to 7–10 and 11–14 years (*p* < 0.001 for all comparisons). A progressive decrease in AC was found in females at the age of 11–14 years compared to the group aged 7–10 years (*p* < 0.05) and in the females aged 15–18 years compared to those aged 7–10 and 11–14 years (*p* < 0.001 for both comparisons).

Regarding the sex differences, males at the age of 15–18 years had significantly higher values of Ep compared to females (*p* ˂ 0.05). The differences in the parameters β, AC, and PWV β were not significant. In the age groups 7–10 and 11–14 years, the carotid wall stiffness parameters were not significantly different between males and females.

The sex- and age-specific distributions of the parameters β, Ep, AC, and PWV β are presented in Table 3.

### 3.3. Carotid Wall Stiffness Indices in the Overweight and Hypertensive Groups Compared to Reference Values

**Figure 2:**
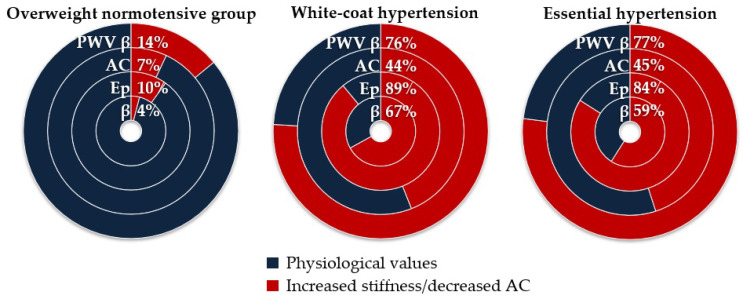
Percentage of overweight normotensive subjects, white coat hypertensives, and essential hypertensives with increased values of carotid stiffness indices or decreased values of carotid compliance compared to the reference values in the healthy non-overweight normotensive population. β, stiffness index beta; Ep, pressure–strain (Peterson’s) elastic modulus; AC, arterial compliance; PWV β, pulse wave velocity.

In the overweight normotensive group (*n* = 69), the carotid stiffness index β was higher than the 95th percentile of the reference sex- and age-specific values in three subjects, Ep in seven subjects, PWV β in ten subjects, and AC was lower than the 5th percentile in five subjects. In subjects with white coat hypertension (*n* = 45), the carotid stiffness index β was higher than the 95th percentile of the reference sex- and age-specific values in 30 subjects, Ep in 40 subjects, PWV β in 34 subjects, and AC was lower than the 5th percentile in 20 subjects. In subjects with essential hypertension (*n* = 44), the carotid stiffness index β was higher than the 95th percentile of reference sex- and age-specific values in 26 subjects, Ep in 37 subjects, PWV β in 34 subjects, and AC was lower than the 5th percentile in 20 subjects (Figure 2).

### 3.4. Regression Analysis of the Effect of Age, Sex, Overweight, and Hypertension

Index β, Ep, AC, and PWV β were strongly associated with age, white coat hypertension, and essential hypertension (*p* < 0.001 for all). The effect of sex was significant only for AC (*p* = 0.006). The effect of overweight was significant for Ep and PWV β (*p* = 0.035, *p* = 0.014, respectively; Table 4).

## 4. Discussion

This study presents pediatric reference values specific to age and sex for carotid stiffness indices β, Ep, AC, and PWV β evaluated using the carotid wall echo-tracking method. Index β, Ep, and PWV β showed a continuous progressive increase in arterial stiffness with age in both sexes. AC showed a continuous progressive decrease in carotid wall compliance in both sexes. The major finding of this study is that both white coat and essential hypertension showed a comparable significant effect on accelerated vascular aging characterized by increased stiffness and decreased carotid wall compliance already in childhood and adolescence. In this study, overweight showed a relatively weak effect on arterial stiffening, which was significant only for PWV β followed by Ep.

The evaluation of the mechanical properties of CCA using the high-resolution echo-tracking method represents a highly accurate tool for evaluating changes in arterial diameter during the cardiac cycle and consequently calculating indices of stiffness and compliance of the arterial wall. With aging, the arteries undergo complex changes in their structure and function resulting from altered cellular mitotic rate and cytokine production, degradation of the extracellular matrix, and the deposition of less elastic structural components [20]. These changes are more evident in the tunica media of the proximal elastic segments and result in a progressive increase in arterial stiffness, which can be accelerated by various cardiovascular risk factors [20,21]. Among these, elevated BP plays a pivotal role in the mechanisms that contribute to early vascular aging [7,22]. For the evaluation of the effect of cardiovascular risk factors and potential arterial damage, it is necessary first to assess the physiological values of the functional and structural parameters of the arterial wall specific to a certain method of examination [23]. In children and adolescents, there are fewer normative tables and lower interobserver correlation between measurements [9,24,25]. Therefore, collecting reference values from different pediatric populations is important for improved precision and broader clinical application.

Regarding the effect of essential hypertension on early vascular aging in pediatric patients, our previous studies showed increased arterial stiffness evaluated using the cardio-ankle vascular index (CAVI), decreased macrovascular endothelial function evaluated using flow-mediated dilation (FMD) at the level of the brachial artery, and decreased microvascular endothelial function evaluated using peripheral arterial tonometry (PAT) in hypertensive children [26,27,28]. In white coat hypertensives, the results were somewhat inconsistent with various examination methods and vascular beds evaluated—FMD showed impaired macrovascular endothelium function comparable with the effect of essential hypertension, CAVI showed an intermediate nonsignificant degree of arterial stiffening, and PAT showed no detectable signs of microvascular endothelial dysfunction [26,27,28]. The present study completes the mosaic of distinct indices of vascular aging studied in pediatric hypertension with a novel finding that both white coat and sustained essential hypertension showed a comparable effect on the echo-tracking parameters of carotid stiffness. Although the long-term consequences of white coat hypertension in childhood remain unclear, there is growing evidence for the associated risk of early target organ damage, such as increased left ventricular mass or intima–media thickness, already in pediatric patients [15,29,30,31,32]. Similarly, the extent of these changes was comparable to the effect of essential hypertension or intermediate between normotensive and hypertensive subjects, and some studies did not find significant differences between white coat hypertensive and normotensive children [15]. However, in adulthood, white coat hypertension was found to be associated with an increased risk of cardiovascular events, cerebrovascular complications, and all-cause mortality [33,34].

The effect of overweight on the parameters of carotid stiffness in this study was detected only using PWV β and Ep and it was much weaker compared to the effect of hypertension. Previously, the parameters β, EP, and PWV β were found to be elevated in obese children compared to a non-obese control group [12]. However, the obese group was also characterized by higher BP levels, and the Ep and PWV β were strongly correlated with systolic pressure in the obese group, but not in the control group. Therefore, a cumulative effect of cardiovascular risk factors could play a role in this result [12]. A meta-analysis of 15 case–control studies in children and adolescents revealed that obesity was associated with an increased carotid and aortic β-stiffness index [13]. Again, the role of confounders, such as elevated BP, was not addressed. In our study, the evaluated group comprised overweight and obese normotensive individuals; therefore, the overall level of adiposity was lower compared to studies with only obese participants. Furthermore, our previous study on arterial stiffness evaluated using CAVI and its more pressure-independent variant CAVI_0_ in overweight hypertensive adolescents showed a certain degree of arterial adaptation to overweight with lower stiffness compared to their normal weight peers [35]. A similar effect was also observed in some studies on arterial stiffness evaluated using central and peripheral PWV in children, adolescents, and young adults [36,37]. In summary, the effect of overweight and obesity on the early vascular aging process in childhood remains unclear. More studies on different indices of arterial characteristics could help find a sensitive marker of the risk of later cardiovascular events related to the effect of overweight in childhood and adolescence.

The clinical significance of our findings is underlined by the fact that the occurrence of cardiovascular risk factors in children and adolescents in developed countries is rapidly increasing. The hard outcomes of these risk factors appear mostly in adulthood; therefore, it is necessary to find noninvasive sensitive tools capable of detecting early preclinical arterial damage [38]. Arterial stiffness is one of the leading markers of the risk related to hypertension. Recently, several therapeutic approaches showed the ability to reduce stiffness beyond passive reductions related to decreased BP, which is expected to confer additional benefits for the clinical outcomes of hypertensive patients [22]. In children, we can expect a greater effect of the non-pharmacological therapeutic interventions on the initial, potentially reversible, alterations of arterial function and structure [39]. Future research on the dynamics of the stiffness parameters over time, frequency of cardiovascular events in later adulthood, and efficacy of preventive interventions could bring important information for more precise risk stratification and improved management of patients with elevated markers of preclinical arteriosclerotic damage. In this study, we focused on the two relatively frequent cardiovascular risk factors in childhood and adolescence—overweight and hypertension. However, other contributing factors, such as dyslipidemia, smoking, insulin resistance, physical inactivity, and their cumulative effect should be clarified in future studies.

Recently, the SARS-CoV-2 pandemic attracted attention to the effect of respiratory infections on cardiovascular health. Generally, infectious diseases are accepted as a significant cardiovascular risk factor, and severe infections in childhood were previously found to be associated with cardiovascular disease in adulthood [40,41]. In adults, the indices of carotid stiffness appear to be sensitive to detect the early adverse effects of both severe and mild respiratory infections already during recovery from the disease [42,43]. Our results could help to study the initial effect of the infections on arterial structure and function in childhood and adolescence.

This study has several limitations. The studied population consisted of solely Caucasian children and adolescents from Slovakia; thus, extrapolation of our findings to other specific populations and regions may be limited. A greater sample size of the overweight and hypertensive groups and a greater proportion of girls in hypertensive groups could improve the generalizability of the findings. The calibration of carotid diameter changes was performed using brachial BP values. This may lead to some overestimation compared to central BP measurements, which seems to be particularly relevant in young individuals [44,45]. The cross-sectional design of the study limits the evaluation of the severity of cardiovascular risk reflected by accelerated stiffening of the carotid wall.

## 5. Conclusions

This study provides pediatric reference values for the echo-tracking parameters of carotid stiffness, which are necessary for their clinical application in the evaluation of the effect of cardiovascular risk factors and potential arterial damage. Index β, Ep, AC, and PWV β were sensitive in detecting the early signs of accelerated vascular aging in children with white coat and essential hypertension. The adverse effect of overweight on carotid stiffness was detected using parameters Ep and PWV β. These findings could help to select the sensitive markers of arteriosclerotic damage under specific clinical conditions in childhood and adolescence and, potentially, help to improve the management of pediatric patients at increased risk of future cardiovascular disease.

## Figures and Tables

**Table 1:** Reference population of healthy non-overweight normotensive children and adolescents.

Age Range	7–10 Years		11–14 Years		15–18 Years	
Total number of subjects	139		133		128	
Number of subjects according to age	7 years: *n* = 32		11 years: *n* = 37		15 years: *n* = 32	
	8 years: *n* = 36		12 years: *n* = 30		16 years: *n* = 30	
	9 years: *n* = 37		13 years: *n* = 32		17 years: *n* = 32	
	10 years: *n* = 34		14 years: *n* = 34		18 years: *n* = 34	
Sex	Males (*n* = 64)	Females (*n* = 75)	Males (*n* = 65)	Females (*n* = 68)	Males (*n* = 59)	Females (*n* = 69)
BMI (kg/m^2^)	15.7 ± 1.6	15.2 ± 1.6	17.8 ± 2.0 ^††^	18.1 ± 2.2 ^††^	20.6 ± 2.0 ^††,‡‡^	20.9 ± 2.1 ^††,‡‡^
Systolic BP (mmHg)	105.3 ± 8.5	105.8 ± 7.4	110.7 ± 14.8 ^††^	109.1 ± 8.4 ^†^	121.7 ± 6.9 ^††, ‡‡^	115.8 ± 9.2 **^,††,‡‡^
Diastolic BP (mmHg)	65.2 ± 7.0	67.4 ± 7.3	65.5 ± 6.0	65.5 ± 7.0	67.3 ± 7.1	69.6 ± 6.9 ^‡‡^
Resting HR (bpm)	77.6 ± 9.7	82.2 ± 10.1 *	73.2 ± 11.7 ^†^	78.1 ± 11.9 *^,†^	67.3± 11.8 ^††,‡^	75.2 ± 11.9 **^,‡‡^

BMI, body mass index; BP, blood pressure; HR, heart rate; bpm, beats per minute. Values are expressed as mean ± SD. * *p* < 0.05; ** *p* < 0.001 compared with males within the same age group. ^†^ *p* < 0.05; ^††^ *p* < 0.001 compared with 7–10 years group within the same sex. ^‡^ *p* < 0.01; ^‡‡^ *p* < 0.001 compared with 11–14 years group within the same sex.

**Table 2:** Clinical data from the reference population, overweight, and hypertensive groups.

	Reference Population	Overweight Normotensives	White Coat Hypertensives	Essential Hypertensives
Number of subjects	400	69	45	44
Sex	188 males 212 females	42 males 27 females	35 males 10 females	40 males 4 females
Age (years)	12.4 ± 3.5	12.8 ± 3.5	16.2 ± 1.8	16.4 ± 1.7
Number of overweight subjects	0/400	69/69	19/45	31/44
BMI (kg/m^2^)	18.0 ± 2.9	23.7 ± 2.8	23.8 ± 3.8	26.3 ± 4.0
Systolic BP (mmHg)	111.1 ± 11.0	118.0 ± 10.4	143.3 ± 12.3	145.0 ± 8.0
Diastolic BP (mmHg)	66.8 ± 7.1	70.0 ± 5.9	73.6 ± 8.3	75.9 ± 8.0
Resting heart rate (bpm)	75.9 ± 12.0	78.7 ± 11.5	75.0 ± 12.3	70.8 ± 11.3

BMI, body mass index; BP, blood pressure; bpm, beats per minute. Values are expressed as mean ± SD.

**Table 3:** Reference values of the carotid wall stiffness indices in healthy non-overweight normotensive children and adolescents.

	7–10 Years		11–14 Years		15–18 Years	
Number of subjects	139		133		128	
	Males (*n* = 64)	Females (*n* = 75)	Males (*n* = 65)	Females (*n* = 68)	Males (*n* = 59)	Females (*n* = 69)
β						
Mean	3.44	3.40	4.13	4.04	4.71	4.54
SD	0.62	0.67	0.43	0.72	0.65	0.50
5th pc	2.50	2.33	3.30	3.27	3.65	3.80
10th pc	2.79	2.50	3.50	3.33	4.04	3.90
50th pc	3.40	3.40	4.20	4.20	4.60	4.50
90th pc	4.20	4.20	4.70	4.60	5.50	5.30
95th pc	4.63	4.48	4.90	4.85	5.86	5.40
Ep (kPa)						
Mean	38.2	38.6	48.3	46.8	57.9	55.1
SD	6.8	7.2	5.7	6.5	8.2	7.3
5th pc	28.0	27.0	40.0	34.9	48.0	45.0
10th pc	30.9	30.0	41.0	38.0	49.0	45.0
50th pc	38.0	39.0	49.0	47.0	57.0	55.0
90th pc	48.1	47.0	55.0	54.0	65.0	65.0
95th pc	49.3	49.0	58.5	55.4	71.7	71.1
AC (mm^2^/kPa)						
Mean	1.37	1.27	1.15	1.16	1.05	1.03
SD	0.27	0.30	0.17	0.20	0.14	0.18
5th pc	1.00	0.75	0.90	0.90	0.83	0.73
10th pc	1.07	0.91	0.91	0.93	0.87	0.81
50th pc	1.34	1.27	1.15	1.16	1.05	1.01
90th pc	1.71	1.60	1.40	1.43	1.20	1.24
95th pc	1.86	1.75	1.46	1.56	1.35	1.40
PWV β (m/s)						
Mean	3.74	3.78	4.17	4.12	4.46	4.46
SD	0.31	0.33	0.24	0.28	0.34	0.30
5th pc	3.27	3.20	3.80	3.50	3.95	4.00
10th pc	3.30	3.30	3.90	3.73	4.10	4.10
50th pc	3.70	3.80	4.10	4.10	4.50	4.40
90th pc	4.10	4.10	4.40	4.50	4.80	4.90
95th pc	4.20	4.20	4.55	4.51	5.12	5.00

β, stiffness index beta; Ep, elastic modulus; AC, arterial compliance; PWV β, pulse wave velocity; SD, standard deviation; pc, percentile.

**Table 4:** Estimated effects of age, sex, overweight, and hypertension on carotid stiffness index β, Ep, AC, and PWV β.

Variable	Coefficient	Standard Error	Units	Probability Value
Number of subjects: 558				
β				
Intercept	2.212	0.120	-	<0.001
Age	0.145	0.009	yr^−1^	<0.001
Male sex (*n* = 305; 55%)	0.044	0.061	-	0.472
Overweight (*n* = 119; 21%)	0.019	0.078	-	0.807
White coat hypertension (*n* = 45; 8%)	1.535	0.115	-	<0.001
Essential hypertension (*n* = 44; 8%)	1.404	0.123	-	<0.001
Ep				
Intercept	19.795	1.502	-	<0.001
Age	2.202	0.112	yr^−1^	<0.001
Male sex (*n* = 305; 55%)	0.278	0.758	-	0.714
Overweight (*n* = 119; 21%)	2.042	0.968	-	0.035
White coat hypertension (*n* = 45; 8%)	28.653	1.439	-	<0.001
Essential hypertension (*n* = 44; 8%)	27.957	1.541	-	<0.001
AC				
Intercept	1.589	0.038	-	<0.001
Age	−0.036	0.003	yr^−1^	<0.001
Male sex (*n* = 305; 55%)	0.054	0.019	-	0.006
Overweight (*n* = 119; 21%)	0.037	0.025	-	0.140
White coat hypertension (*n* = 45; 8%)	−0.221	0.037	-	<0.001
Essential hypertension (*n* = 44; 8%)	−0.249	0.040	-	<0.001
PWV β				
Intercept	3.041	0.058	-	<0.001
Age	0.088	0.004	yr^−1^	<0.001
Male sex (*n* = 305; 55%)	−0.045	0.029	-	0.125
Overweight (*n* = 119; 21%)	0.092	0.037	-	0.014
White coat hypertension (*n* = 45; 8%)	0.831	0.055	-	<0.001
Essential hypertension (*n* = 44; 8%)	0.839	0.059	-	<0.001

β, stiffness index beta; Ep, pressure–strain (Peterson’s) elastic modulus; AC, arterial compliance; PWV β, pulse wave velocity. Parameters of male sex, overweight, white coat hypertension, and essential hypertension are dummy-coded.

## Data Availability

Data will be available on request to the corresponding author.
